# Improved Assessment of Orbital Stability of Rhythmic Motion with Noise

**DOI:** 10.1371/journal.pone.0119596

**Published:** 2015-03-23

**Authors:** Jooeun Ahn, Neville Hogan

**Affiliations:** 1 Department of Mechanical Engineering, University of Victoria, Victoria, British Colombia, Canada; 2 Department of Mechanical Engineering, Massachusetts Institute of Technology, Cambridge, Massachusetts, United States of America; 3 Department of Brain and Cognitive Sciences, Massachusetts Institute of Technology, Cambridge, Massachusetts, United States of America; University of California, Merced, UNITED STATES

## Abstract

Mathematical techniques have provided tools to quantify the stability of rhythmic movements of humans and machines as well as mathematical models. One archetypal example is the use of Floquet multipliers: assuming periodic motion to be a limit-cycle of a nonlinear oscillator, local stability has been assessed by evaluating the rate of convergence to the limit-cycle. However, the accuracy of the assessment in experiments is questionable: Floquet multipliers provide a measure of orbital stability for deterministic systems, but various components of biological systems and machines involve inevitable noise. In this study, we show that the conventional estimate of orbital stability, which depends on regression, has bias in the presence of noise. We quantify the bias, and devise a new method to estimate orbital stability more accurately. Compared with previous methods, our method substantially reduces the bias, providing acceptable estimates of orbital stability with an order-of-magnitude fewer cycles.

## Introduction

Rhythmic processes are common in nature and technology. An accurate empirical measure of their stability is important, but in many fields, the number of periods that can be observed to form the basis of that measure may be quite limited in practice. For example, fluctuations in populations and ecosystems are often periodic (consider the 17-year cicada) but the number of cycles that may be observed is necessarily limited. In robotic engineering, recent research has focused on legged locomotion, progressing from hexapods [1] to quadrupeds [2, 3] and bipeds [4–6]. Again, the number of steps that can reliably be observed is limited, especially early in technology development, when instability is most prevalent and assessment of stability is most important. When we consider the interaction of robots with humans to aid recovery after neurological injury such as stroke [7, 8] the challenge is even more severe. The stability of neurologically-impaired subjects’ walking may be an important measure of their degree of functional recovery but, due to injury, they are usually incapable of walking for extended periods, even with assistance. In this paper, we show that a limited window of observation has a profound influence on the accuracy of conventional stability estimates. We propose an alternative that provides unbiased estimates of the stability of rhythmic processes while requiring a significantly reduced number of cycles.

One important measure of stability that has received considerable attention is the Floquet multiplier. As a branch of the theory of ordinary differential equations, Floquet introduced a theorem that established the stability of a periodic solution of a linear system [9]. Studies in many fields (motor neuroscience, biomechanics, robotics, ecology etc.), address rhythmic stability using this Floquet multiplier [10–20]. However, it is important to recognize that the original Floquet theory was designed for a deterministic and linear system. In contrast, typical systems of interest (humans, robots, ecosystems, etc.) involve highly nonlinear dynamics and stochastic noise. Nevertheless, the Floquet multiplier has been seamlessly extended to nonlinear systems. The local stability of a limit cycle of a nonlinear system may be assessed by the Floquet multiplier of the linearized *return map* or *Poincaré map* [21–23]. Once a Poincaré section, a subset of state-space which defines the end or beginning of one cycle, is chosen, the Poincaré map between the states at the current cycle (**x**_k_) and the next cycle (**x**_k+1_) can be defined; **x**_k+1_ = P(**x**_k_). A limit cycle is a fixed point (x*) of the Poincaré map that satisfies x* = P(x*). If all the eigenvalues of the linearized map evaluated at the fixed point—the Floquet multipliers—have magnitude less than one, the limit cycle is asymptotically stable. If any of the Floquet multipliers has magnitude larger than one, the limit cycle is unstable. Smaller Floquet multipliers indicate faster convergence to a fixed point or stronger stability.

This linearized stability analysis requires an assumption that the perturbation is small enough such that higher-order nonlinear terms are negligible compared to the linearized dynamics. In most cases of experimental studies to measure orbital stability, this assumption is acceptable; we usually let a system repeat a cycle without severe perturbation when we want to assess its orbital stability. Even if we confine our interest to local stability, the conventional Floquet multiplier measure may not be applicable to a system with stochastic noise; as we show below, the ubiquitous noise in biological, robotic or ecological systems distorts estimates of the Floquet multiplier. At a minimum, the validity of conventional estimates of Floquet multipliers in the presence of stochastic noise should be established. If a sufficiently large dataset can average out the effect of stochastic noise, it is important to identify the minimum dataset size that enables a reasonably accurate measure.

Previous studies used a least-squares linear fit of the relation between adjacent cycles to assess the orbital stability of rhythmic movements that include unpredictable variability [10, 11, 18–20]. To estimate the Floquet multipliers of human walking, Hurmuzlu and Basdogan searched for the best affine fit relating the state of every stride with the state of the next stride [10]. Dingwell and Kang used a similar approach but they additionally assumed that the average trajectory was the unperturbed limit cycle [11]. However, to the best of our knowledge, none of prior experimental studies validated the accuracy of the estimated Floquet multipliers in the presence of noise.

In this study, we show that previous estimates of orbital stability, based on linear regression, are biased due to noise and the limited set of data. By quantifying the bias, we propose a new method that reduces the number of cycles required to obtain unbiased orbital stability by more than an order of magnitude.

## Methods

### Mathematical Description

As an initial study, we address the simplest case of a one-dimensional return map. The linearized return map in a one-dimensional state space can be expressed as

$$s_{k+1}-s^{*}=\lambda (s_{k}-s^{*}),$$

where *λ* is the Floquet multiplier. Assuming that stochastic noise is added on each cycle as a random variable,

$$s_{k+1}-s^{*}=\lambda (s_{k}-s^{*})+\delta_{k+1},$$

where *δ*_*k*_ is from a distribution with zero mean and standard deviation of *σ*_*δ*_. The noise in the current cycle is assumed to be independent of the noise in the previous or next cycle. Substituting *x*_*k*_ for *s*_*k*_—s*, the time series of interest can be defined in the following recursive formula:

x1=δ1, andxk+1=λxk+δk+1,orxk=∑i=1kλk-iδi (1)

By this description, the error dynamics of a system that has a stable periodic solution and stochastic noise is equivalent to an autoregressive (AR) process of order one. Finding the Floquet multiplier consists of estimating the constant *λ*, which describes the best linear map between input **y** = {*x*_1_, *x*_2_, *x*_3_, …, *x*_*n*-1_} and output **z** = {*x*_2_, *x*_3_, *x*_4_, …, *x*_*n*_}.

### Bias of Previous Methods

The bias of previous methods to estimate *λ* was addressed by numerical simulation. With a fixed value of the Floquet multiplier, *λ*, the time series {*x*_*k*_} was constructed by adding noise. The length of the time series, *n* varied from 10 to 1000. Orbital stability was estimated by linear regression following the methods used in previous experimental studies [10, 11]. The simulation and estimation were repeated 1000 times for each *n*. The bias between the true *λ*, and the mean of the 1000 estimated values, $\hat{\lambda }$ was evaluated for each *n*. We tested *λ* of 0.75, 0.5 and 0.25. We investigated the effect of three types of the noise distribution—normal, uniform, and asymmetric lognormal.

The accuracy of the estimate was statistically addressed by testing a null hypothesis, H_0_: the estimated orbital stability comes from a normal distribution with mean equal to the actual value. Rejection of H_0_ indicated that the estimation method was not acceptable. For each *n*, a t-test was performed at a significance level of 5%. Numerical simulation and analysis was implemented in Matlab (Mathworks Inc.).

We also addressed the bias analytically. The assumption that the noise in each cycle is an independent random variable allowed us to approximate the expected bias in a simple closed form.

### Review of Standard Methods for AR models

Because the time series of Equation (1) is an AR process, we investigated whether standard identification methods for AR models could eliminate or minimize the bias. We ran the same simulations and estimated the Floquet multiplier using the Yule-Walker equation [24, 25] and Burg’s method [26] to assess bias due to these standard methods. The simulation was implemented in Matlab (Mathworks Inc.) using the Signal Processing Toolbox.

### An Improved Method to Assess a Floquet Multiplier

An analytical expression for the bias was developed which enabled us to propose an improved method to assess local orbital stability. We derived an expression for the bias of the Yule-Walker equation, and then added an adjustment to reduce the bias. The adjustment required knowledge of the actual value of the Floquet multiplier. As a reasonable approximation we used the estimate derived from Burg’s method to substitute for the actual value. We evaluated the accuracy of this new method in the same way we tested the accuracy of previous methods: with a fixed value of the Floquet multiplier, *λ*, the time series {*x*_*k*_} were constructed by adding noise, where the length of the time series, *n* varied from 10 to 1000. The orbital stability was estimated by our new method, and the simulation was repeated 1000 times for each *n*. The bias between the true *λ*, and the mean of the 1000 estimated values,$\hat{\lambda }$ was evaluated and the null hypothesis H_0_ was tested for each *n*.

## Results

**Fig 1:**
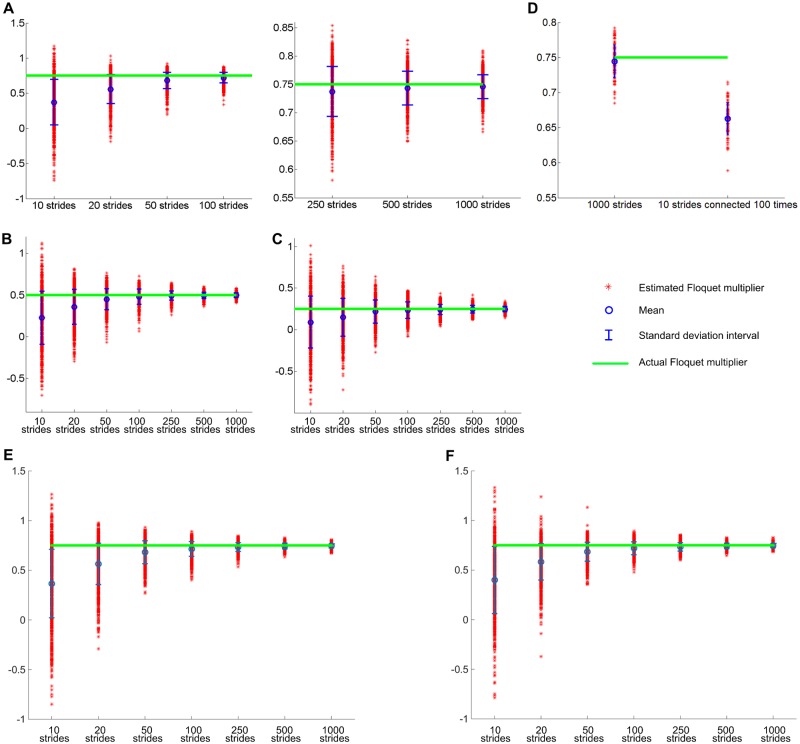
Estimation of a Floquet multiplier (FM) using linear regression. Time series with different length, *n* were generated using Equation (1). Noise from a normal distribution was added to each cycle and values of FM were 0.75 (A), 0.5 (B) and 0.25 (C). The FM was evaluated for each time series using linear regression. The simulation was repeated 1000 times for each *n*. Red asterisks show the 1000 estimates of FM, blue circles denote their mean and the whiskers denote their standard deviation. For any value of actual FM, the mean of the estimated FMs is always smaller, though the discrepancy decreases as *n* increases. For clarity, case A is presented in two panels, the second with an expanded vertical scale. H_0_—the null hypothesis that the estimated orbital stability comes from a normal distribution with mean equal to the actual value—is rejected even when *n* = 1000. In D, the estimation of FM = 0.75 from a time series with *n* = 1000 was compared with estimation from a stitched time series that consisted of 100 time series with *n* = 10. Though the length of the two cases are the same (1000), the stitched time series has significantly larger bias than a single time series. All of these results remain similar when the noise comes from a uniform or lognormal distribution; the estimation of FM using linear regression shows similar bias in the presence of noise from a uniform distribution (E), or an asymmetric lognormal distribution (F).

We found that the conventional methods yield a substantial bias: linear regression overestimates the strength of stability by underestimating the magnitude of the Floquet multiplier. Fig. 1 shows the simulation results. The distribution of the estimated Floquet multiplier,$\hat{\lambda }$ always has a smaller mean than the actual Floquet multiplier, *λ*. Though the accuracy of the estimate improves with an increasing number of strides, the convergence is unacceptably slow. The null hypothesis, H_0_, that the estimated orbital stability comes from a normal distribution with mean equal to the actual value was rejected even when *n* = 1000. (The normality of the distribution of the estimation was investigated using the Lilliefors test, and the p-value of the normality test was close to or greater than 0.01, suggesting that the deviation from normal distribution is arguably marginal, and t-test can be used.) This significant bias is not affected either by the type of noise distribution or by the noise level; results remain similar whether the noise comes from a normal, uniform, or asymmetric lognormal distribution, and whether the noise level is high or low as long as the noise is not zero.

Analysis explains this bias. Following the methods in previous experimental studies [10, 11], the linear regression or least square fit estimates the Floquet multiplier as

$$\hat{\lambda }=\frac{\sum_{i=1}^{n-1}\left(x_{i}-\bar{x})(y_{i}-\bar{y}\right)}{\sum_{i=1}^{n-1}{\left(x_{i}-\bar{x}\right)}^{2}},$$ (2)

where $\bar{x}$ and $\bar{y}$ are the mean of {*x*_1_, *x*_2_, *x*_3_, …, *x*_*n*-1_} and {*x*_2_, *x*_3_, *x*_4_, …, *x*_*n*_} respectively, and *y*_*i*_ *= λx*_*i*_ *+ δ*_*i+1*_ from (1). Then, the expectation of the bias becomes

$$E(\hat{\lambda }-\lambda )=E\left(\frac{\sum_{i=1}^{n-1}\left\{(x_{i}-\bar{x})(y_{i}-\bar{y})-\lambda {\left(x_{i}-\bar{x}\right)}^{2}\right\}}{\sum_{i=1}^{n-1}{\left(x_{i}-\bar{x}\right)}^{2}}\right).$$ (3)

Using a property of AR process, and assuming a large enough number of cycles, the above expectation approximates the following:

$$E(\hat{\lambda }-\lambda )≅-\frac{1+\lambda }{n-1}\left(1-\frac{1-\lambda^{n-1}}{\left(n-1)(1-\lambda \right)}\right).$$ (4)

A detailed derivation is in S1 Appendix. Equation (4) explains the finite bias, which decreases as the length of the time series increases: with large enough *n*, the expectation of the bias is roughly proportional to 1/*n*. Note that this bias does not depend on *σ*_*δ*_, or the noise level.

**Fig 2:**
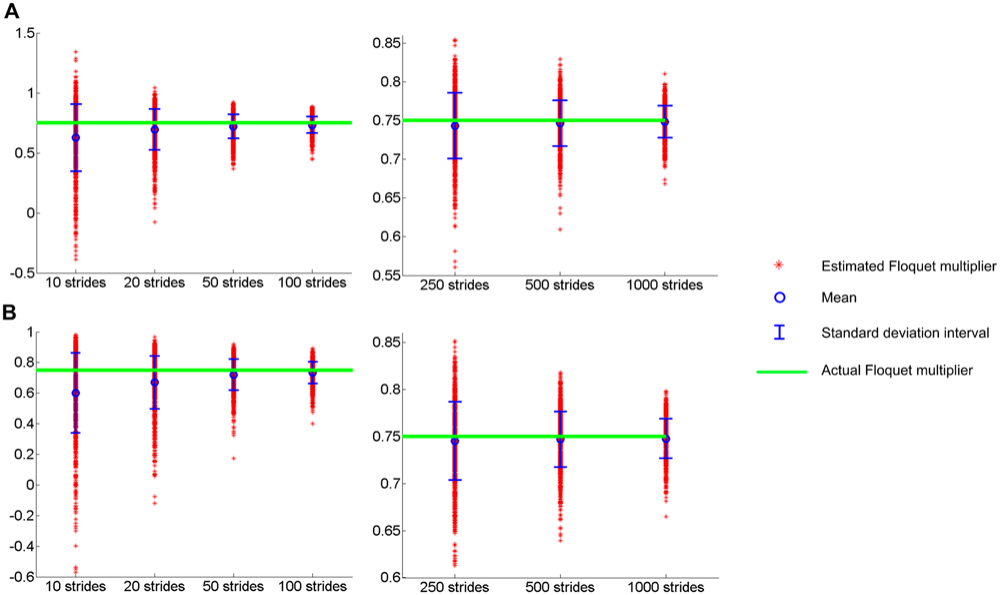
Estimation of a Floquet multiplier (FM) using the Yule-Walker equation and Burg’s Method. Time series with different length, *n* were generated with FM = 0.75. The FM was estimated for each time series using the Yule-Walker equation (A), and Burg’s method (B). In each case, the simulation was repeated 1000 times for each *n*. For both methods, the mean of the estimated FMs is always smaller than the actual FM though the discrepancy decreases as *n* increases. H_0_—the null hypothesis that the estimated orbital stability comes from a normal distribution with mean equal to the actual value—is rejected even when *n* = 1000. Noise drawn from normal, uniform and lognormal distributions yield similar results.

Standard methods to assess the stability of AR processes fare little better. Fig. 2 shows the bias of standard methods used for AR processes—the Yule-Walker equation [24, 25] and Burg’s method [26]. Both provide better measures of the Floquet multiplier, but they still show bias, which is more prominent when *n* is small. Previous studies have addressed the limitations of the Yule-Walker equation [27, 28], and claimed that Burg’s method can resolve some of them. However, as shown in Fig. 2, Burg’s method could not eliminate the bias either, though it provides better estimation, confining the estimated Floquet multiplier between -1 and 1.

None of these methods accepts H_0_ even when *n* = 1000. To recap, the methods used in previous experimental studies [10, 11] as well as standard methods for AR processes underestimate the magnitude of the Floquet multiplier and accordingly overestimate the strength of orbital stability.

We devised a more accurate measure of orbital stability by quantifying the amount of bias. Considering that the Yule-Walker equation and Burg’s method result in relatively smaller bias, and the Yule-Walker equation for an AR process is simpler, we added an adjustment to the Yule-Walker equation. Estimation of the Floquet multiplier from the Yule-Walker equation is

$${\hat{\lambda }}_{YW}=\frac{\sum_{i=1}^{n-1}x_{i}x_{i+1}}{\sum_{i=1}^{n-1}x_{i}{}^{2}}.$$ (5)

Using properties of AR processes and assuming a sufficiently large number of cycles, the expected bias is approximately

$$E({\hat{\lambda }}_{YW}-\lambda )≅-\frac{\lambda^{3}-\lambda^{2n+1}}{\left(n-1)(1-\lambda^{2}\right)}.$$ (6)

Again, the detailed derivation is in S1 Appendix. As in the case of Equation (5), the expected bias is approximately proportional to 1/*n*, but does not depend on the noise level, *σ*_*δ*_. Based on Equation (6), we propose a new estimator:

$${\hat{\lambda }}_{new}={\hat{\lambda }}_{YW}+\frac{{\hat{\lambda }}_{Burg}{}^{3}-{\hat{\lambda }}_{Burg}{}^{2n+1}}{\left(n-1)(1-{\hat{\lambda }}_{Burg}{}^{2}\right)},$$ (7)

where ${\hat{\lambda }}_{Burg}$ is the estimate from Burg’s method. The true *λ* on the right hand side of Equation (6) is unknown and therefore is replaced with an estimate. For the adjustment term, ${\hat{\lambda }}_{Burg}$ is chosen because Burg’s method guarantees stability of the estimated AR model, and it is most reliable when orbital stability is close to marginal [27]. On the other hand, the uncorrected estimate is based on the Yule-Walker equation because its simplicity enables ready derivation of the bias and any necessary adjustment in closed form.

**Fig 3:**
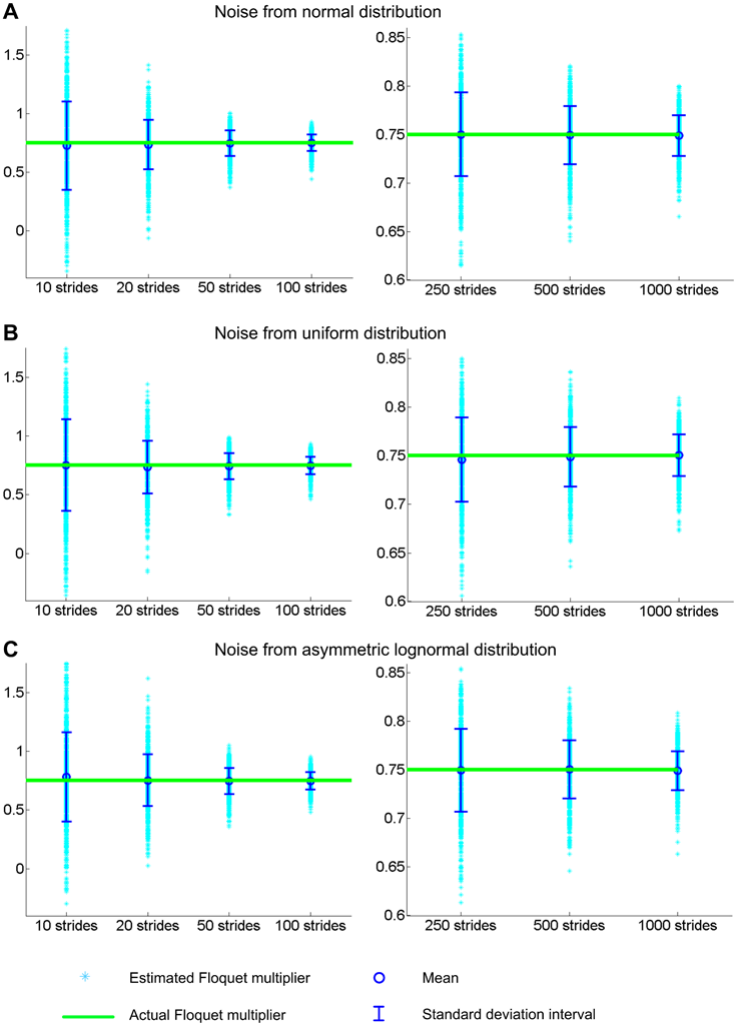
Estimation of a Floquet multiplier (FM) using a new method that estimates the bias of previous methods. An adjustment calculated based on Burg’s method was added to a first estimate based on the Yule-Walker equation. Time series with different length, *n* were generated with FM = 0.75 and various distributions of noise: normal (A), uniform (B) and asymmetric lognormal (C). The FM was evaluated for each time series using this new method. The simulation was repeated 1000 times for each *n*. Compared with the previous methods, the bias is substantially reduced. With this method, H_0_—the null hypothesis that the estimated orbital stability comes from a distribution with mean equal to the actual value—is accepted when *n* ≥ 50 for normally or uniformly distributed noise (A and B), and *n* ≥ 20 for asymmetrically distributed noise (C).

Fig. 3 shows the distribution of Floquet multipliers estimated by our new method. The bias is significantly reduced. Statistical analysis now accepts the null hypothesis H_0_ when *n* = 50 or larger. This substantial improvement is not affected either by the type of noise distribution or by the noise level; similar results are obtained whether the noise comes from a normal, a uniform, or an asymmetric lognormal distribution, and whether the noise level is high or low as long as the noise is not zero.

## Discussion

In previous studies, the orbital stability of rhythmic behavior has been assessed by estimating Floquet multipliers. However, the original Floquet theory assumed deterministic evolution, whereas practical systems include inevitable stochastic noise. Here, we showed that previous measures of orbital stability are significantly biased by noise. We quantified the bias and devised a substantially better method to assess orbital stability.

### Previous measures are biased

Our simulation results show that conventional methods based on regression always underestimate the Floquet multiplier, and therefore overestimate orbital stability (Fig. 1). The bias is particularly prominent when the length of the time series, *n* is small. This trend is due to uncorrelated noise tending to “whiten” a time series. When *n* is small, the cumulative effect of orbital stability is weak, and the effect of noise is strong. Significant noise obscures the intrinsic linear correlation between input and output of the return map so that linear regression indicates a weaker correlation or, equivalently, a smaller slope.

Considering the result of Fig. 1, it is clear that previous measures of the orbital stability of human walking are biased. The Floquet multiplier of human walking was estimated from 20 m walking [10], and 200 m walking [11], which approximately correspond to 10 and 100 strides, respectively. But based on our simulations, the bias is substantial when *n* = 10 (50%) or even when *n* = 100 (5%). In fact, the null hypothesis that the estimated Floquet multiplier comes from a normal distribution with mean equal to the actual value is rejected even when *n* = 1000.

### A common fix doesn’t help

Simulation results (Fig. 1) and mathematical analysis (Equation (5)) show clearly that repetitions of measures with small *n* cannot substitute for a single measure with large *n*. Because observing a large number of cycles reduces bias, it may seem reasonable to construct a long time-series by “stitching” short time-series together. Unfortunately, this does not work. Fig. 1-D shows that connecting many short time series to generate a long time series is not a solution. Though concatenating 100 time-series with *n* = 10 results in a better estimate than the short time-series with *n* = 10, it still shows substantial bias compared to a single time-series with *n* = 1000. Concatenation increases the length of the time series and therefore improves the reliability of regression. However, it is still deficient in the information to accurately measure orbital stability. Physically, orbital stability determines how much of the perturbed dynamics remains effective in the following cycles. If the periodic motion is weakly stable, a large portion of the perturbed dynamics will be retained in the next cycle. The concatenated time series lacks the effect of the first perturbation on the 11^th^, 12^th^, 13^th^, …, and 1000^th^ cycles; the effect of the second perturbation on the 12^th^, 13^th^, …, and 1000^th^ cycles; and so on. All of these long-retained and accumulated effects, which enhance the accuracy of regression, are available only in a long time-series that is obtained from a single trial without a break.

These observations indicate a significant challenge in the design of experiments with human subjects. In practice, performing 1000 or more cycles of rhythmic movement is extremely demanding. For example, 1000 strides of adult walking approximately correspond to a mile of distance traversed. In principle, the bias in measures of orbital stability might be reduced by instructing subjects to execute a large number of uninterrupted cycles, but (even if they consented) that would likely induce significant side-effects due to, e.g. fatigue. This problem becomes much more pressing if we attempt to measure the orbital stability of patients’ movements in clinical studies.

An even subtler challenge arises if we attempt to measure orbital stability to assess performance in motor learning studies. In that case, orbital stability should be measured before it changes due to adaptation or learning induced by a large number of repetitions. Here we reported a method that can produce an estimate with minimal or negligible bias with as few as 50 cycles.

### The bias and adjustment do not depend on noise level or distribution

We derived an analytical expression of the bias. The derivation was simplified by an assumption that the noise in the current cycle is independent of the noise in the neighboring cycles. Neither the specific profile of the probability density function nor the level of the noise affects the resulting bias.

The robustness of bias against the noise level further underlines the problem of previous measures. Equation (5) and (6) clarify that the bias is insensitive to noise level, *σ*_*δ*_ as long as the noise level is not zero. Therefore, it is not valid to claim that the orbital stability measured from young and healthy subjects is more reliable than that from old or injured subjects. The bias needs to be addressed and adjusted even though the experimental observation involves relatively less noise.

Bias that is independent of the level of noise and its distribution also significantly facilitates the application of our improved method. Because the bias is induced by noise, it may seem necessary to identify the noise properties to assess and eliminate the bias. However, our analysis shows that we do not need detailed knowledge of the noise process to estimate the bias as long as the noise has no correlation. This implies that we can apply same methods for various systems with various levels and distributions of noise without additional effort to identify the noise properties. For example, this enables us to address the bias of orbital stability of normal walking and pathological walking using the same method.

### Limitations

Our analysis assumed that the noise added to each trial has no correlation. Therefore, additional consideration is necessary when applying our method to a system with anti-correlated or correlated noise. While the assumption of uncorrelated noise is generally accepted (particularly in human motor execution) and is supported by some experimental observations [29], other studies also showed non-zero correlation in the variability of motor output. For example, stride intervals in human walking and heart beat exhibit long range correlations, suggesting that the variability in those motor behaviors cannot be modeled as white noise [30, 31]. However, we need to distinguish the variability of the observed motor output from the assumed noise that causes the variability. In Equation (1), the variability of *x* may exhibit correlation even if *δ* has no correlation. In fact, a recent study showed that the long-range correlations observed in the stride intervals of human walking and other rhythmic motor behavior may be explained by uncorrelated noise filtered through stable rhythmic dynamics of sensory-motor systems [32]. The observed correlated variability in motor output does not necessarily invalidate the assumption of uncorrelated noise, which our analysis is built on. The assumption that the original unfiltered source of variability is white noise is common and highly effective in control and signal processing theory as well as in computational biology. It is also the basis of the widely-applicable Kalman filter [33].

For this initial study, our analysis used a simple one-dimensional model. Generalization from this model to multi-dimensional models is deferred to future work. On the other hand, our linear model is a one-dimensional description of any linearized model with stochastic noise per cycle.

Rhythmic dynamics are ubiquitous in various kinds of systems, and an accurate measure of orbital stability is therefore critical in numerous fields. For linear systems, the orbital stability assessed by a Floquet multiplier establishes global stability of periodic motion. For general nonlinear systems, the Floquet multiplier quantifies how fast the system recovers from small perturbations. Here, we showed that previous empirical estimates of orbital stability did not properly take account of the effect of noise. We quantified the bias due to noise and developed a new method that can provide an unbiased estimate of orbital stability within a reasonably small number of cycles. This is particularly important for experiments with human subjects or clinical evaluation of patients to avoid serious side effects due to fatigue or the unwanted effect of motor learning.

## Supporting Information

S1 Appendix This appendix presents detailed derivations of Equation (5) and (6). (PDF)
