# Supplementary material for: Improved Assessment of Orbital Stability of Rhythmic Motion with Noise
Source: PLoS One. 2015 Mar 23;10(3):e0119596. doi: 10.1371/journal.pone.0119596 (PMC4370583; doi:10.1371/journal.pone.0119596)
Supplement: S1 Appendix — (PDF) [file pone.0119596.s001.pdf]

Appendix S1

Bias of Floquet Multiplier Estimation by Linear Regression

The linear regression or least square fit estimates the Floquet multiplier as

$$\hat{\lambda} = \frac{\sum_{i=1}^{n-1}(x_i - \bar{x})(y_i - \bar{y})}{\sum_{i=1}^{n-1}(x_i - \bar{x})^2},$$

where $\bar{x}$ and $\bar{y}$ are the mean of $\{x_1, x_2, x_3, \ldots, x_{n-1}\}$ and $\{x_2, x_3, x_4, \ldots, x_n\}$ respectively, and $y_i = \lambda x_i + \delta_{i+1}$. The expectation of the bias becomes

$$E(\hat{\lambda} - \lambda) = E\left(\frac{\sum_{i=1}^{n-1}\left\{(x_i - \bar{x})(y_i - \bar{y}) - \lambda(x_i - \bar{x})^2\right\}}{\sum_{i=1}^{n-1}(x_i - \bar{x})^2}\right). \tag{S1}$$

Assuming a stable periodic process, or $|\lambda| < 1$, the AR process becomes stationary and thus has finite mean and variance, satisfying

$$E(x_{i+1}) = E(y_i) = E(x_i), \text{ and}$$

$$\text{var}(x_{i+1}) = \text{var}(y_i) = \text{var}(x_i).$$

The mean of x_i, or $\bar{x}$ is the solution of

$$E(x_{i+1}) = \lambda E(x_i) - E(\delta_{i+1}) = \lambda E(x_i) = E(x_i), \text{ which is zero.}$$

Therefore, re-writing the equation for the variance,

$$\sigma_x^{\ 2} = E(x_{i+1}^{\ 2}) - E(x_{i+1})^2 = E(x_i^{\ 2}) - E(x_i)^2, \text{ or}$$

$$\sigma_x^{\ 2} = E(x_{i+1}^{\ 2}) = E((\lambda x_i + \delta_{i+1})^2) = \lambda^2 E(x_i^{\ 2}) + 2\lambda E(x_i \delta_{i+1}) + E(\delta_{i+1}^{\ 2}) = E(x_i^{\ 2}).$$

By definition, x_i is a weighted sum of $\delta_1, \delta_2, \delta_3, \ldots, \delta_i$, none of which is correlated with δ_{i+1}. Therefore, $E(x_i \delta_{i+1})$ becomes zero. Then,

$$\sigma_x^{\ 2} = \lambda^2 E(x_i^{\ 2}) + E(\delta_{i+1}^{\ 2}) = \lambda^2 \sigma_x^{\ 2} + \sigma_\delta^{\ 2}, \text{ or } \sigma_x^{\ 2} = \frac{\sigma_\delta^{\ 2}}{1 - \lambda^2},$$

where σ_δ is the standard deviation of the noise, δ_k. Therefore, assuming a large enough number of cycles, the denominator of (S1) becomes

$$\sum_{i=1}^{n-1}(x_i - \bar{x})^2 = (n-1)\sigma_x^{\ 2} \cong \frac{(n-1)\sigma_\delta^{\ 2}}{1 - \lambda^2}. \tag{S2}$$

Using (S1) and (S2), the expectation of bias can be approximated as

$$E(\hat{\lambda} - \lambda) \cong \frac{1 - \lambda^2}{(n-1)\sigma_\delta^{\ 2}} E\left(\sum_{i=1}^{n-1}\left\{(x_i - \bar{x})(y_i - \bar{y}) - \lambda(x_i - \bar{x})^2\right\}\right). \tag{S3}$$

Using $y_i = \lambda x_i + \delta_{i+1}$,

$$\begin{aligned}
&\sum_{i=1}^{n-1}\left\{(x_i-\bar{x})(y_i-\bar{y})-\lambda(x_i-\bar{x})^2\right\} \\
&=\sum_{i=1}^{n-1}\left\{(x_i-\bar{x})(\lambda x_i+\delta_{i+1}-\bar{y})-\lambda(x_i^{\,2}-2x_i\bar{x}+\bar{x}^2)\right\} \\
&=\sum_{i=1}^{n-1}\left\{x_i\delta_{i+1}-x_i\bar{y}+x_i\bar{x}\lambda-\bar{x}\delta_{i+1}+\overline{xy}-\lambda\bar{x}^2\right\} \\
&=\sum_{i=1}^{n-1}x_i\delta_{i+1}-(\bar{y}-\lambda\bar{x})\sum_{i=1}^{n-1}x_i-\bar{x}\sum_{i=1}^{n-1}\delta_{i+1}+(n-1)(\overline{xy}-\lambda\bar{x}^2).
\end{aligned} \tag{S4}$$

By definition of $\bar{x}$, the sum $\sum_{i=1}^{n-1}x_i$ can be re-written as $(n-1)\bar{x}$. Therefore, from (S4),

$$\begin{aligned}
&\sum_{i=1}^{n-1}\left\{(x_i-\bar{x})(y_i-\bar{y})-\lambda(x_i-\bar{x})^2\right\} \\
&=\sum_{i=1}^{n-1}x_i\delta_{i+1}-(n-1)(\overline{xy}-\lambda\bar{x}^2)-\bar{x}\sum_{i=1}^{n-1}\delta_{i+1}+(n-1)(\overline{xy}-\lambda\bar{x}^2) \\
&=\sum_{i=1}^{n-1}x_i\delta_{i+1}-\bar{x}\sum_{i=1}^{n-1}\delta_{i+1}.
\end{aligned} \tag{S5}$$

From (S3) and (S5),

$$E(\hat{\lambda}-\lambda)\cong\frac{1-\lambda^2}{(n-1)\sigma_\delta^{\,2}}E\left(\sum_{i=1}^{n-1}x_i\delta_{i+1}-\bar{x}\sum_{i=1}^{n-1}\delta_{i+1}\right). \tag{S6}$$

By definition, δ_p and δ_q are independent when $p \neq q$. Therefore,

$$E(\delta_p\ \delta_q) = 0 \text{ if } p \neq q \tag{S7}$$

because

$$\int_{-\infty}^{\infty}\int_{-\infty}^{\infty}\delta_p\delta_q f(\delta_p)f(\delta_q)d\delta_p d\delta_q=\int_{-\infty}^{\infty}\delta_q\left(\int_{-\infty}^{\infty}\delta_p f(\delta_p)d\delta_p\right)d\delta_q=0.$$

Note that the validity of (S7) does not depend on the specific shape of the distribution; the probability density function, *f*, can be any function as long as the mean of the noise is zero. Whether the distribution is symmetric like a normal and uniform distribution or asymmetric like a lognormal distribution, (S7) remains valid.

Now note that x_i is a weighted sum of δ_1, δ_2, δ_3, … δ_i, by definition in (1). Therefore, $E(x_i\delta_{i+1}) = 0$ for each *i*, and $E\left(\sum_{i=1}^{n-1}x_i\delta_{i+1}\right)=0$. From (S6),

$$E(\hat{\lambda}-\lambda)\cong-\frac{1-\lambda^2}{(n-1)\sigma_\delta^{\,2}}E\left(\bar{x}\sum_{i=1}^{n-1}\delta_{i+1}\right). \tag{S8}$$

By definition,

$$
\begin{aligned}
x_1 &= \delta_1 \\
x_2 &= \lambda\delta_1 + \delta_2 \\
x_3 &= \lambda^2\delta_1 + \lambda\delta_2 + \delta_3 \\
&\vdots \qquad\qquad\qquad \ddots \\
x_{n-1} &= \lambda^{n-2}\delta_1 + \lambda^{n-3}\delta_2 + \cdots + \delta_{n-1}.
\end{aligned}
$$

Therefore,

$$
\begin{aligned}
\bar{x} &= \frac{1}{(n-1)}(x_1 + x_2 + x_3 + \cdots + x_{n-1}) \\
&= \frac{1}{(n-1)}\left(\frac{1-\lambda^{n-1}}{1-\lambda}\delta_1 + \frac{1-\lambda^{n-2}}{1-\lambda}\delta_2 + \cdots + \frac{1-\lambda^2}{1-\lambda}\delta_{n-2} + \delta_{n-1}\right).
\end{aligned} \tag{S9}
$$

From (S8) and (S9),

$$
E(\hat{\lambda}-\lambda) \cong -\frac{1-\lambda^2}{(n-1)^2\sigma_\delta{}^2} E\left(\left(\frac{1-\lambda^{n-1}}{1-\lambda}\delta_1 + \frac{1-\lambda^{n-2}}{1-\lambda}\delta_2 + \cdots + \delta_{n-1}\right)(\delta_2 + \delta_3 + \cdots + \delta_n)\right).
$$

By (S7), any term with $\delta_p\ \delta_q$ ($p \neq q$) does not contribute to the expectation, and only terms with $\delta_p{}^2$ remain. Therefore,

$$
E(\hat{\lambda}-\lambda) \cong -\frac{1-\lambda^2}{(n-1)^2\sigma_\delta{}^2} E\left(\frac{1-\lambda^{n-2}}{1-\lambda}\delta_1{}^2 + \frac{1-\lambda^{n-3}}{1-\lambda}\delta_2{}^2 + \cdots + \delta_{n-1}{}^2\right). \tag{S10}
$$

By definition, $E(\delta_p{}^2) = \sigma_\delta{}^2$, and (S10) becomes

$$
\begin{aligned}
E(\hat{\lambda}-\lambda) &\cong -\frac{1-\lambda^2}{(n-1)^2}\left(\frac{1-\lambda^{n-2}}{1-\lambda} + \frac{1-\lambda^{n-3}}{1-\lambda} + \cdots + \frac{1-\lambda^2}{1-\lambda} + 1\right) \\
&= -\frac{1-\lambda^2}{(n-1)^2}\left(\frac{(n-2)-(\lambda+\lambda^2+\cdots+\lambda^{n-2})}{1-\lambda}\right) \\
&= -\frac{1-\lambda^2}{(n-1)^2}\left(\frac{(n-1)-(1+\lambda+\lambda^2+\cdots+\lambda^{n-2})}{1-\lambda}\right) \\
&= -\frac{1+\lambda}{(n-1)}\left(1-\frac{1-\lambda^{n-1}}{(n-1)(1-\lambda)}\right).
\end{aligned} \tag{S11}
$$

Bias of Floquet Multiplier Estimation by the Yule-Walker Equation

The Yule-Walker equation estimates the Floquet multiplier as

$$\hat{\lambda}_{YW} = \frac{\sum_{i=1}^{n-1} x_i x_{i+1}}{\sum_{i=1}^{n-1} x_i^{\,2}}. \tag{S12}$$

Following the same procedure that derived above (S2), the variance of x_i, $\sigma_x^{\,2}$ becomes

$$\sigma_x^{\,2} = \frac{\sigma_\delta^{\,2}}{1-\lambda^2},$$

where σ_δ is the standard deviation of the noise, δ_k. Assuming a large enough number of cycles, the denominator of (S12) approximates

$$\sum_{i=1}^{n-1} x_i^{\,2} \cong \frac{(n-1)\sigma_\delta^{\,2}}{1-\lambda^2}. \tag{S13}$$

By definition of x_i, the numerator of (S12) becomes

$$\sum_{i=1}^{n-1} x_i x_{i+1} = \sum_{i=1}^{n-1} \left(\lambda^{i-1}\delta_1 + \lambda^{i-2}\delta_2 + \cdots + \delta_i\right)\left(\lambda^{i}\delta_1 + \lambda^{i-1}\delta_2 + \cdots + \delta_{i+1}\right). \tag{S14}$$

From (S13) and (S14),

$$E(\hat{\lambda}_{YW} - \lambda) \cong \frac{1-\lambda^2}{(n-1)\sigma_\delta^{\,2}} E\left(\sum_{i=1}^{n-1} \left(\lambda^{i-1}\delta_1 + \lambda^{i-2}\delta_2 + \cdots + \delta_i\right)\left(\lambda^{i}\delta_1 + \lambda^{i-1}\delta_2 + \cdots + \delta_{i+1}\right)\right) - \lambda$$

.

By (S7), any term with $\delta_p\ \delta_q$ ($p \neq q$) does not contribute to the expectation, and only terms with $\delta_p^{\,2}$ remain. Therefore,

$$E(\hat{\lambda}_{YW} - \lambda) \cong \frac{1-\lambda^2}{(n-1)\sigma_\delta^{\,2}} E\left(\sum_{i=1}^{n-1} \left(\lambda^{2i-1}\delta_1^{\,2} + \lambda^{2i-3}\delta_2^{\,2} + \cdots + \lambda\delta_i^{\,2}\right)\right) - \lambda. \tag{S15}$$

Expanding what is inside Σ,

$$\begin{aligned}
i = 1:&\quad \lambda\delta_1^{\,2} \\
i = 2:&\quad \lambda^3\delta_1^{\,2} + \lambda\delta_2^{\,2} \\
i = 3:&\quad \lambda^5\delta_1^{\,2} + \lambda^3\delta_2^{\,2} + \lambda\delta_3^{\,2} \\
\vdots &\qquad\qquad\qquad\qquad \ddots \\
i = n-1:&\ \lambda^{2n-3}\delta_1^{\,2} + \lambda^{2n-5}\delta_2^{\,2} + \cdots + \lambda\delta_{n-1}^{\,2}.
\end{aligned}$$

Calculating the sum,

$$\sum_{i=1}^{n-1} \left(\lambda^{2i-1}\delta_1^{\,2} + \lambda^{2i-3}\delta_2^{\,2} + \cdots + \lambda\delta_i^{\,2}\right) = \frac{\lambda - \lambda^{2n-1}}{1-\lambda^2}\delta_1^{\,2} + \frac{\lambda - \lambda^{2n-3}}{1-\lambda^2}\delta_2^{\,2} + \cdots + \frac{\lambda - \lambda^3}{1-\lambda^2}\delta_{n-1}^{\,2}.$$

Therefore, using $E(\delta_p^{\,2}) = \sigma_\delta^2$, (S15) becomes

$$E(\hat{\lambda}_{YW} - \lambda) \cong \frac{1-\lambda^2}{(n-1)\sigma_\delta^{\,2}} \left(\frac{(n-1)\lambda - (\lambda^{2n-1} + \lambda^{2n-3} + \cdots + \lambda^3)}{1-\lambda^2}\right)\sigma_\delta^{\,2} - \lambda, \text{ or}$$

$$E(\hat{\lambda}_{YW}) \cong \lambda - \frac{\lambda^3 - \lambda^{2n+1}}{(n-1)(1-\lambda^2)}. \tag{S16}$$
